# Response to discrepancies in publications related to HMB-FA and ATP supplementation

**DOI:** 10.1186/s12986-017-0227-x

**Published:** 2017-11-28

**Authors:** Jacob M Wilson

**Affiliations:** The Applied Science & Performance Institute, Tampa, United States

**Keywords:** Hypertrophy, Training adaptations, Muscle growth, Strength

## Abstract

Gentles and Phillips have submitted questions regarding three recent papers investigating ATP, HMB and the combination. The questions pertaining to the homogeneity of subjects’ characteristics between the three different published papers, and why there appears to be differences in the number of subjects in placebo groups across studies. This response addresses each of these issues and demonstrates that there are no discrepancies between papers but rather a misunderstanding of the papers previously published.

Dear Dr. Xu Lin, Liang Sun and Associate Editors,

Recently, Gentles and Phillips [1] wrote a communication requesting further explanations regarding our recent publications on ATP [2], HMB-FA [3] and co-ingestion of ATP and HMB-FA [4] on training adaptations, resulting from the same study with the clinical trial identifier: NCT01508338. We acknowledge that the authors have invested a great deal of time following our work on multiple platforms, and our reexamination only serves to further our understanding of the significance of these studies. In their effort to better understand our research, Gentles and Phillips [1] have submitted questions pertaining to the homogeneity of subjects’ characteristics between the three different published papers, and why there appears to be differences in the number of subjects in placebo groups across studies [2–4]. Our response addresses each of these issues and demonstrates that there are no discrepancies between papers but rather a misunderstanding of the papers previously published.

Gentles and Phillips [1] question the homogeneity between papers, providing a table showing that subject characteristics for Wilson et al. [2] and Wilson et al. [3] are even identical. The table by Gentles and Phillips is incorrect. The correct participant characteristics in Wilson et al. [2] are as follows: age 23.4 ± 0.7 with 1-RM of 1.71 ± 0.04, 1.34 ± 0.03 and 2.05 ± 0.04 times body weight for squat, bench press, and deadlift, respectively. We are uncertain as to where the numbers presented by Gentles and Phillips came from in their Table 1. We have provided the corrected Table 1 below. The values in the corrected table add a degree of heterogeneity which may reasonably be expected, but are still quite homogenous. This was accomplished by the randomization procedure. We arranged the subjects by strength, and then performed the randomization.

**Table 1:** Sample means and standard deviations

	Wilson et al. according to [1] Entire group (*n* = 21)	Actual Wilson et al. [2] Entire group (*n* = 21)	Wilson et al. [3] Entire group (*n* = 20)	Lowery et al. [4] Entire group (*n* = 17)
Age (years)	21.6 ± 0.5	23.4 ± 0.7	21.6 ± 0.5	21.7 ± 0.4
1RM squat^a^	1.7 ± 0.04	1.71 ± 0.04	1.7 ± 0.04	1.7 ± 0.07
1RM bench press^a^	1.3 ± 0.04	1.34 ± 0.03	1.3 ± 0.04	1.3 ± 0.05
1RM deadlift^a^	2.0 ± 0.05	2.05 ± 0.04	2.0 ± 0.05	2.0 ± 0.06

^a^Mean 1RM values are expressed relative to body mass

Gentles and Phillips also question the supplement protocol for the placebo group, allowing for the use of the same control group for all three publications from the same study. Subjects received three times per day a gel pack consisting of 1 g of HMB-FA, or a matching placebo gel pack; and once per day a capsule either containing 400 mg per day of Disodium ATP or a matching placebo. Subjects consumed a gel pack and the capsule 30 min before exercise as well as 1 gel pack before lunch and 1 before dinner. On the non-training days, participants consumed 1 gel pack and the capsule 30 min before breakfast and 1 additional gel pack with lunch and dinner. The daily supplementation protocols, 3 gel packs and 1 capsule, were identical in each group. The HMB-FA + ATP publication [4] describes the full supplementation protocol. For clarity, the ATP [2] and HMB-FA [3] publications describe the administered supplements relevant for the data being presented. The information for the ATP group provided in the clinical trial registration is updated and reflects the correct supplement protocol.

Lastly, Gentles and Phillips would like further clarification regarding the differences in the number of subjects in placebo groups across studies, 12 recruited, 3 drop-outs (2 due to injury, 1 due to time constraints), 9 completed for HMB-FA [3] and HMB-FA + ATP [4], and the 10 placebo-supplemented subjects completing the ATP study [2]. The ATP trial was completed in a later semester and thus, two additional subjects were recruited to the placebo group to blind the treatments. One of the additional placebo subjects was lost due to injury. Due to the cost and high intensity nature of the protocol, we felt it ethically correct to utilize placebo subjects from the original HMB-FA and HMB-FA + ATP cohort in addition to the ATP-supplemented subjects and the placebo subject added. The total number of subjects recruited for the ATP study was twenty-six, 14 in the placebo (4 total drop-outs, 3 due to injury, 1 due to time constraints) and 12 in the ATP group (1 drop-out due to injury).

We hope this serves to clear up any misinterpretation in our studies.

Sincerely,

Jacob Wilson, PhD

Corresponding Author

## Abbreviations

ATP: Adenosine triphosphate
HMB-FA: beta-Hydroxy beta-methylbutyric free acid
